# On Distribution Reduction and Algorithm Implementation in Inconsistent Ordered Information Systems

**DOI:** 10.1155/2014/307468

**Published:** 2014-08-28

**Authors:** Yanqin Zhang

**Affiliations:** School of Economics, Xuzhou Institute of Technology, Xuzhou, Jiangsu 221008, China

## Abstract

As one part of our work in ordered information systems, distribution reduction is studied in inconsistent ordered information systems (OISs). Some important properties on distribution reduction are studied and discussed. The dominance matrix is restated for reduction acquisition in dominance relations based information systems. Matrix algorithm for distribution reduction acquisition is stepped. And program is implemented by the algorithm. The approach provides an effective tool for the theoretical research and the applications for ordered information systems in practices. For more detailed and valid illustrations, cases are employed to explain and verify the algorithm and the program which shows the effectiveness of the algorithm in complicated information systems.

## 1. Introduction

In Pawlak's original rough set theory [1], partition or equivalence (indiscernibility) is an important and primitive concept. However, partition or equivalence relation, as the indiscernibility relation in Pawlak's original rough set theory, is still restrictive for many applications. To address this issue, several interesting and meaningful extensions to equivalence relation have been proposed in the past, such as neighborhood operators [2], tolerance relations [3], and others [4–10]. Moreover, the original rough set theory does not consider attributes with preference ordered domain, that is, criteria. In many real life practices, we often face problems in which the ordering of properties of the considered attributes plays a crucial role. One such type of problem is the ordering of objects. For this reason, Greco et al. proposed an extension rough set theory, called the dominance based rough set approach (DRSA), to take into account the ordering properties of criteria [11–16]. This innovation is mainly based on substitution of the indiscernibility relation by a dominance relation. Moreover, Greco et al. characterizes the DRSA and decision rules induced from rough approximations, while the usefulness of the DRSA and its advantages over the CRSA (classical rough set approach) are presented [11–16]. In DRSA, condition attributes are criteria and classes are preference ordered. Several studies have been made about properties and algorithmic implementations of DRSA [10, 17–19].

Nevertheless, only a limited number of methods using DRSA to acquire knowledge in inconsistent ordered information systems have been proposed and studied. Pioneering work on inconsistent ordered information systems with the DRSA has been proposed by Greco et al. [11–16], but they did not clearly point out the semantic explanation of unknown values. Shao and Zhang [20] further proposed an extension of the dominance relation in incomplete ordered information systems. Their work was established on the basis of the assumption that all unknown values are lost. Despite this, they did not mention the underlying concept of attribute reduction in inconsistent ordered decision system but they mentioned an approach to attribute reduction in consistent ordered information systems. Therefore, the purpose of this paper is to develop approaches to attribute reductions in inconsistent ordered information systems (IOIS). In this paper, theories and approaches of distribution reduction are investigated in inconsistent ordered information systems. Furthermore, algorithm of matrix computation of distribution reduction is introduced, from which we can provide a new approach to attributes reductions in inconsistent ordered information systems.

The rest of this paper is organized as follows. Some preliminary concepts are briefly recalled in Section 2. In Section 3, theories and approaches of distribution reduction are investigated in IOIS. In Section 4, we restate the definition of dominance matrix in ordered information systems and step the matrix algorithm for distribution reduction acquisition. Preparations are implemented to place the algorithm and the program is designed. The algorithm and the corresponding program we designed can provide a tool to theoretical research and applications of criterion based information system. Cases are employed to illustrate the algorithm and the program in Section 5. It is shown that the algorithm and program are effective in complicated information system. Furthermore conclusions on what we study in this paper are drawn to understand this paper briefly.

## 2. Ordered Information Systems

The following recalls necessary concepts and preliminaries required in the sequel of our work. Detailed description of the theory can be found in [11–16].

An information system with decisions is an ordered quadruple *I* = (*U*, *A* ∪ *D*, *F*, *G*), where *U* = {*x*_1_, *x*_2_,…, *x*_*n*_} is a nonempty finite set of objects; *A* ∪ *D* is a nonempty finite attributes set; *A* = {*a*_1_, *a*_2_,…, *a*_*p*_} denotes the set of condition attributes; *D* = {*d*_1_, *d*_2_,…, *d*_*q*_} denotes the set of decision attributes, *A*∩*D* = *ϕ*; *F* = {*f*_*k*_∣*U* → *V*_*k*_, *k* ≤ *p*}, *f*_*k*_(*x*) is the value of *a*_*k*_ on *x* ∈ *U*; *V*_*k*_ is the domain of *a*_*k*_, *a*_*k*_ ∈ *A*; *G* = {*g*_*k*′_∣*U* → *V*_*k*′_, *k*′ ≤ *q*}, *g*_*k*′_(*x*) is the value of *d*_*k*′_ on *x* ∈ *U*,  *V*_*k*′_ is the domain of *d*_*k*′_, *d*_*k*′_ ∈ *D*. In an information system, if the domain of an attribute is ordered according to a decreasing or increasing preference, then the attribute is a criterion. An information system is called an ordered information system (OIS) if all condition attributes are criterions.

Assume that the domain of a criterion *a* ∈ *A* is completely preordered by an outranking relation ⪰_*a*_; then *x*⪰_*a*_ *y* means that *x* is at least as good as *y* with respect to criterion *a*. And we can say that *x* dominates *y*. In the following, without any loss of generality, we consider condition and decision criterions having a numerical domain; that is, *V*_*a*_⊆*R* (*R* denotes the set of real numbers).

We define *x*⪰*y* by *f*(*x*, *a*) ≥ *f*(*y*, *a*) according to increasing preference, where *a* ∈ *A* and *x*, *y* ∈ *U*. For a subset of attributes *B*⊆*A*, *x*⪰_*B*_ *y* means that *x*⪰_*a*_ *y* for any *a* ∈ *B*. That is to say *x* dominates *y* with respect to all attributes in *B*. Furthermore, we denote *x*⪰_*B*_ *y* by *xR*_*B*_^≥^ *y*. In general, we indicate an ordered information system with decision by *I* ^⪰^ = (*U*, *A* ∪ *D*, *F*, *G*). Thus the following definition can be obtained.

Let *I* ^⪰^ = (*U*, *A* ∪ *D*, *F*, *G*) be an ordered information system with decisions, for *B*⊆*A*; denote

(1) RB⪰={(xi,xj)∈U×U ∣ fl(xi)≥fl(xj),∀al∈B};RD⪰={(xi,xj)∈U×U ∣ gm(xi)≥gm(xj),∀dm∈D}.

*R*_*B*_^⪰^ and *R*_*D*_^⪰^ are called dominance relations of information system *I* ^⪰^.

If we denote

(2) [xi]B⪰={xj∈U ∣ (xj,xi)∈RB⪰}={xj∈U ∣ fl(xj)≥fl(xi),∀al∈B},[xi]D⪰={xj∈U ∣ (xj,xi)∈RD⪰}={xj∈U ∣ gm(xj)≥gm(xi),∀dm∈D},

then the following properties of a dominance relation are trivial.

Let *R*_*A*_^⪰^ be a dominance relation. The following properties hold.

1. *R*_*A*_^⪰^ is reflexive and transitive, but not symmetric, so it is not an equivalence relation.
2. If *B*⊆*A*, then *R*_*A*_^⪰^⊆*R*_*B*_^⪰^.
3. If *B*⊆*A*, then [*x*_*i*_]_*A*_^⪰^⊆[*x*_*i*_]_*B*_^⪰^.
4. If *x*_*j*_ ∈ [*x*_*i*_]_*A*_^⪰^, then [*x*_*j*_]_*A*_^⪰^⊆[*x*_*i*_]_*A*_^⪰^ and [*x*_*i*_]_*A*_^⪰^ = ∪{[*x*_*j*_]_*A*_^⪰^∣*x*_*j*_ ∈ [*x*_*i*_]_*A*_^⪰^}.
5. [*x*_*j*_]_*A*_^⪰^ = [*x*_*i*_]_*A*_^⪰^ if and only if *f*(*x*_*i*_, *a*) = *f*(*x*_*j*_, *a*)  (∀*a* ∈ *A*).
6. *J* = ∪{[*x*]_*A*_^⪰^∣*x* ∈ *U*} constitute a covering of *U*.

For any subset *X* of *U*, and *A* of *I* ^⪰^, define

(3) RA⪰_(X)={x∈U[x]A⪰⊆X},RA⪰¯(X)={x∈U ∣ [x]A⪰∩X≠ϕ}.

$\underline{R_{A}^{⪰}}(X)$ and $\bar{R_{A}^{⪰}}(x)$ are said to be the lower and upper approximations of *X* with respect to a dominance relation *R*_*A*_^⪰^. And the approximations have also some properties which are similar to those of Pawlak approximation spaces.

For an ordered information system with decisions *I* ^⪰^ = (*U*, *A* ∪ *D*, *F*, *G*), if *R*_*A*_^⪰^⊆*R*_*D*_^⪰^, then this information system is consistent, otherwise, this information system is inconsistent (IOIS).

**Example 1 .**

An ordered information system is given in Table 1.

From the table, we have

(4) [x1]A⪰={x1,x2,x5,x6};  [x2]A⪰={x2,x5,x6};[x3]A⪰={x2,x3,x4,x5,x6};  [x4]A⪰={x4,x6};[x5]A⪰={x5};  [x6]A⪰={x6};[x1]d⪰=[x5]d⪰={x1,x5};[x2]d⪰=[x4]d⪰={x1,x2,x4,x5};[x3]d⪰=[x6]d⪰={x1,x2,x3,x4,x5,x6}.

Obviously, by the above, we have *R*_*A*_^⪰^⊈*R*_*d*_^⪰^, so the system in Table 1 is inconsistent.

For a simple description, the following information system with decisions is based on dominance relations, that is, ordered information system.

## 3. Theories of Distribution Reduction in Inconsistent Ordered Information Systems

Let *I* ^⪰^ = (*U*, *A* ∪ *D*, *F*, *G*) be an information system with decisions, and *R*_*B*_^⪰^,  *R*_*D*_^⪰^ dominance relations derived from condition attributes set *B*⊆*A* and decision attributes set *D*, respectively. For *B*⊆*A*, denote

(5) $$\begin{array}{c} \frac{U}{R_{B}^{⪰}}=\left\{{\left[x_{i}\right]}_{B}^{⪰}\;|\;x_{i}\in U\right\},\\ \frac{U}{R_{d}^{⪰}}=\left\{D_{1},D_{2},\ldots ,D_{r}\right\},\\ \mu_{B}^{⪰}\left(x\right)=\left(\frac{\left|D_{1}\cap {\left[x\right]}_{B}^{⪰}\right|}{\left|U\right|},\frac{\left|D_{2}\cap {\left[x\right]}_{B}^{⪰}\right|}{\left|U\right|},\ldots ,\frac{\left|D_{r}\cap {\left[x\right]}_{B}^{⪰}\right|}{\left|U\right|}\right),\\ \gamma_{B}^{⪰}\left(x\right)=\max\left\{\frac{\left|D_{1}\cap {\left[x\right]}_{B}^{⪰}\right|}{\left|U\right|},\frac{\left|D_{2}\cap {\left[x\right]}_{B}^{⪰}\right|}{\left|U\right|},\ldots ,\frac{\left|D_{r}\cap {\left[x\right]}_{B}^{⪰}\right|}{\left|U\right|}\right\}, \end{array}$$

where [*x*]_*B*_^⪰^ = {*y* ∈ *U*∣(*x*, *y*) ∈ *R*_*B*_^⪰^}. Furthermore, we let *μ*_*B*_^⪰^(*x*) be a distribution function about attributions set *B* and *γ*_*B*_^⪰^(*x*) maximum distribution function about attributions set *B*.

**Definition 2 .**

Let *α* = (*a*_1_, *a*_2_,…, *a*_*n*_) and *β* = (*b*_1_, *b*_2_,…, *b*_*n*_) be two vectors with *n* dimensions. If *a*_*i*_ = *b*_*i*_,  (*i* = 1,2,…, *n*), we say that *α* is equal to *β* and is denoted by *α* = *β*. If *a*_*i*_ ≤ *b*_*i*_,  (*i* = 1,2,…, *n*), we say that *α* is less than *β* and is denoted by *α* ≤ *β*. Otherwise, if it exists *i*_0_  (*i*_0_ ∈ {1,2,…, *n*}) such that *a*_*i*_0__ > *b*_*i*_0__, we say *α* is not less than *β* and it is denoted by *α*≰*β*,

such as (1,2, 3)≰(1,1, 4) and (1,1, 4)≰(1,2, 3).

From the above, we can have the following propositions immediately.

**Proposition 3 .**

Let *I* ^⪰^ = (*U*, *A* ∪ *D*, *F*, *G*) be an inconsistent information system.

1. If *B*⊆*A*, then *μ*_*A*_^⪰^(*x*) ≤ *μ*_*B*_^⪰^(*x*), ∀*x* ∈ *U*.
2. If *B*⊆*A*, then *γ*_*A*_^⪰^(*x*) ≤ *γ*_*B*_^⪰^(*x*), ∀*x* ∈ *U*.
3. If [*y*]_*B*_^⪰^⊇[*x*]_*B*_^⪰^, then *μ*_*B*_^⪰^(*y*) ≤ *μ*_*B*_^⪰^(*x*), ∀*x*, *y* ∈ *U*.
4. If [*y*]_*B*_^⪰^⊇[*x*]_*B*_^⪰^, then *γ*_*B*_^⪰^(*y*) ≤ *γ*_*B*_^⪰^(*x*), ∀*x*, *y* ∈ *U*.

**Definition 4 .**

Let *I* ^⪰^ = (*U*, *A* ∪ *D*, *F*, *G*) be an inconsistent information system. If *μ*_*B*_^⪰^(*x*) = *μ*_*A*_^⪰^(*x*), for all *x* ∈ *U*, we say that *B* is a distribution consistent set of *I* ^⪰^. If *B* is a distribution consistent set, and no proper subset of *B* is a distribution consistent set, then *B* is called a distribution consistent reduction of *I* ^⪰^.

**Definition 5 .**

Let *I* ^⪰^ = (*U*, *A* ∪ *D*, *F*, *G*) be an inconsistent information system. If *γ*_*B*_^⪰^(*x*) = *γ*_*A*_^⪰^(*x*), for all *x* ∈ *U*, we say that *B* is a maximum distribution consistent set of *I* ^⪰^. If *B* is a maximum distribution set, and no proper subset of *B* is a maximum distribution consistent set, then *B* is called a maximum distribution consistent reduction of *I* ^⪰^.

**Example 6 .**

For the system in Table 1, if we denote

(6) $$\begin{array}{c} D_{1}={\left[x_{1}\right]}_{d}^{⪰}={\left[x_{5}\right]}_{d}^{⪰},\\ D_{2}={\left[x_{2}\right]}_{d}^{⪰}={\left[x_{4}\right]}_{d}^{⪰},\\ D_{3}={\left[x_{3}\right]}_{d}^{⪰}={\left[x_{6}\right]}_{d}^{⪰}, \end{array}$$

then we can have

(7) $$\begin{array}{c} \mu_{A}^{⪰}\left(x_{1}\right)=\left(\frac{1}{3},\frac{1}{2},\frac{2}{3}\right);\\ \mu_{A}^{⪰}\left(x_{2}\right)=\left(\frac{1}{6},\frac{1}{3},\frac{1}{2}\right);\\ \mu_{A}^{⪰}\left(x_{3}\right)=\left(\frac{1}{6},\frac{1}{2},\frac{5}{6}\right);\\ \mu_{A}^{⪰}\left(x_{4}\right)=\left(0,\frac{1}{6},\frac{1}{3}\right);\\ \mu_{A}^{⪰}\left(x_{5}\right)=\left(\frac{1}{6},\frac{1}{6},\frac{1}{6}\right);\\ \mu_{A}^{⪰}\left(x_{6}\right)=\left(0,0,\frac{1}{6}\right),\\ \gamma_{A}^{⪰}\left(x_{1}\right)=\frac{2}{3};\;\;\gamma_{A}^{⪰}\left(x_{2}\right)=\frac{1}{2};\;\;\gamma_{A}^{⪰}\left(x_{3}\right)=\frac{5}{6};\\ \gamma_{A}^{⪰}\left(x_{4}\right)=\frac{1}{3};\;\;\gamma_{A}^{⪰}\left(x_{5}\right)=\frac{1}{6};\;\;\gamma_{A}^{⪰}\left(x_{6}\right)=\frac{1}{6}. \end{array}$$

When *B* = {*a*_2_, *a*_3_}, it can be easily checked that [*x*]_*A*_^⪰^ = [*x*]_*B*_^⪰^, for all *x* ∈ *U*, so that*μ*_*B*_^⪰^(*x*) = *μ*_*A*_^⪰^(*x*) and *γ*_*B*_^⪰^(*x*) = *γ*_*A*_^⪰^(*x*) are true and *B* = {*a*_2_, *a*_3_} is a distribution consistent set of *I* ^⪰^. Furthermore, we can examine that {*a*_2_} and {*a*_3_} are not consistent sets of *I* ^⪰^. That is to say *B* = {*a*_2_, *a*_3_} is a distribution reduction and is a maximum distribution reduction of *I* ^⪰^.

Moreover, it can easily be calculated that *B*′ = {*a*_1_, *a*_3_} and *B*′′ = {*a*_1_, *a*_2_} are not distribution consistent sets of *I* ^⪰^. Thus there exist only one distribution reduction and maximum distribution reduction of *I* ^⪰^ in the system of Table 1, which are {*a*_2_, *a*_3_}.

The distribution consistent set and the maximum distribution consistent set are related in the following theorem.

**Theorem 7 .**

Let *I* ^⪰^ = (*U*, *A* ∪ *D*, *F*, *G*) be an ordered information system and *B*⊆*A* is a distribution consistent set of *I* ^⪰^ if and only if *B* is a maximum distribution consistent set of *I* ^⪰^.

**Proof**

It can be proved immediately from corresponding definitions and properties. From the definitions of distribution and maximum distribution consistent set, the key results of the implication is that [*x*]_*B*_^⪰^ = [*x*]_*A*_^⪰^ always holds for any *x* ∈ *U* while *B* is a distribution consistent set or maximum distribution consistent set. Thus, the theorem can be acquired immediately.

**Theorem 8 .**

Let *I* ^⪰^ = (*U*, *A* ∪ *D*, *F*, *G*) be an ordered information system.

 
*P* : *B*⊆*A* is a distribution consistent set of *I* ^⪰^.
 
*Q*: While *μ*_*A*_^⪰^(*y*)≰*μ*_*A*_^⪰^(*x*), [*y*]_*B*_^⪰^⊈[*x*]_*B*_^⪰^ holds for any *x*, *y* ∈ *U*.

Then we have *P*⇒*Q*.

**Proof**

We will prove  ¬*Q*⇒¬*P*. Assume that when *μ*_*A*_^⪰^(*y*)≰*μ*_*A*_^⪰^(*x*), [*y*]_*B*_^⪰^⊈[*x*]_*B*_^⪰^ does not hold and that implies [*y*]_*B*_^⪰^⊆[*x*]_*B*_^⪰^. So we can obtain *μ*_*B*_^⪰^(*y*) ≤ *μ*_*B*_^⪰^(*x*) by Proposition 3(3). On the other hand, since *B* is a distribution consistent set of *I* ^⪰^, we have *μ*_*A*_^⪰^(*x*) = *μ*_*B*_^⪰^(*x*) and *μ*_*A*_^⪰^(*y*) = *μ*_*B*_^⪰^(*y*). Hence we can get *μ*_*A*_^⪰^(*y*) ≤ *μ*_*A*_^⪰^(*x*), which is a contradiction. The theorem is proved.

The distribution consistent set requires that the classification ability of the consistent remains the same with the original data table. That is, *B* ⊂ *A*, which is a distribution consistent set of *A*, must satisfy the fact that [*x*]_*B*_^⪰^ = [*x*]_*A*_^⪰^ holds for any *x* ∈ *U*. This is very strict and other reductions studied in [21] may not reach this special condition.

## 4. Matrix Algorithm for Distribution Reduction Acquisition in Inconsistent Ordered Information Systems

In this section, the dominance matrices will be put as a restatement and matrices will be employed to realize the calculation of distribution reductions.

**Definition 9 .**

Let *I* ^⪰^ = (*U*, *A* ∪ *D*, *F*, *G*) be an ordered information system, and *B* ⊂ *A*. Denote

(8) $$\begin{array}{c} M_{B}={\left(m_{ij}\right)}_{n\times n},\;\text{where}\;\;m_{ij}=\{\begin{array}{cc} 1, & x_{j}\in {\left[x_{i}\right]}_{B}^{⪰},\\ 0, & \text{otherwise}. \end{array} \end{array}$$

The matrix *M*_*B*_ is called dominance matrix of attributes set *B*⊆*A*. If |*B*| = *l*, we say that the order of *M*_*B*_ is *l*.

**Definition 10 .**

Let *I* ^⪰^ = (*U*, *A* ∪ *D*, *F*, *G*) be an ordered information system and *M*_*B*_ and *M*_*C*_ are dominance matrices of attributes sets *B*, *C*⊆*A*. The intersection of *M*_*B*_ and *M*_*C*_ is defined by

(9) $$\begin{array}{c} M_{B}\cap M_{C}={\left(m_{ij}\right)}_{n\times n}\cap {\left(m_{ij}^{\prime }\right)}_{n\times n}={\left(\min\left\{m_{ij},m_{ij}^{\prime }\right\}\right)}_{n\times n}. \end{array}$$

The intersection defined above can be implemented by the operator *“*.∗*”* in Matlab platform, *M*_*B*_∩*M*_*C*_ = *M*_*B*_.∗*M*_*C*_, that is, the product of elements in corresponding positions. Then the following properties are obvious.

**Proposition 11 .**

Let *M*_*B*_, *M*_*C*_ be dominance matrices of attributes sets *B*, *C*⊆*A*; the following results always hold.

1. *m*_*ii*_ = 1.
2. *M*_*B*∪*C*_ = *M*_*B*_∩*M*_*C*_.

From the above, we can see that a dominance relation of objects has one-one correspondence to a dominance matrix. The combination of dominance relations can be realized by the corresponding matrices and the dominance relations can be compared by the corresponding matrices from the following definitions.

**Definition 12 .**

Let *M*_*A*_ = (*α*_1_, *α*_2_,…, *α*_*n*_)^*T*^ and *M*_*B*_ = (*β*_1_, *β*_2_,…, *β*_*n*_)^*T*^ be matrices with *n* × *n* dimensions and *α*_*i*_ and *β*_*i*_ row vectors, respectively. If *α*_*i*_ ≤ *β*_*i*_ holds, for any *i* ≤ *n*, we say that *M*_*A*_ is less than *M*_*B*_ and it is denoted by *M*_*A*_ ≤ *M*_*B*_.

By the definitions, dominance matrices have the following properties straightly.

**Proposition 13 .**

Let *I* ^⪰^ = (*U*, *A* ∪ *D*, *F*, *G*) be an ordered information system and *B*⊆*A*. The dominance matrices with respect to *A* and *B* are, respectively, *M*_*A*_ and *M*_*B*_. Then *M*_*A*_ ≤ *M*_*B*_.

In the following, we give the preparation of matrix computation for distribution reductions in ordered information systems.

**Proposition 14 .**

Let *I* ^⪰^ = (*U*, *A* ∪ *D*, *F*, *G*) be an ordered information system and *U* = {*x*_1_, *x*_2_,…, *x*_*n*_} and *A* = {*a*_1_, *a*_2_,…, *a*_*p*_}. Then

(10) $$\begin{array}{c} M_{A}=\overset{p}{\underset{i=1}{⋂}}M_{\left\{a_{i}\right\}}=\left(\begin{array}{cccc} a_{11} & a_{12} & \cdots & a_{1n}\\ a_{21} & a_{22} & \cdots & a_{2n}\\ \vdots & \vdots & \ddots & \vdots \\ a_{n1} & a_{n2} & \cdots & a_{nn} \end{array}\right) \end{array}$$

and any vector *α*_*i*_ = (*a*_*i*1_, *a*_*i*2_,…, *a*_*in*_) represents the dominance class of object *x*_*i*_ by the values 0 and 1, where 0 means the object not included in the class and 1 means the object included in the class.

**Theorem 15 .**

Let *I* ^⪰^ = (*U*, *A* ∪ *D*, *F*, *G*) be an ordered information system and *B*⊆*A*. *B* is a consistent set if and only if *M*_*B*_ = M_*A*_.

**Proof**

As is known, [*x*]_*A*_^⪰^⊆[*x*]_*B*_^⪰^ holds since *B*⊆*A*.

(⇒) For *B* is a distribution consistent set, one can have*μ*_*B*_ = *μ*_*A*_. Then, for any *x* and *D*_*j*_, we have |*D*_*j*_∩[*x*]_*A*_^⪰^ | = |*D*_*j*_∩[*x*]_*B*_^⪰^|. Since [*x*]_*A*_^⪰^⊆[*x*]_*B*_^⪰^, it is obvious that [*x*]_*A*_^⪰^ = [*x*]_*B*_^⪰^. That is, the row vectors in *M*_*B*_ and *M*_*A*_ are correspondingly the same. Then *M*_*B*_ = *M*_*A*_.

(⇐) Since *M*_*B*_ = *M*_*A*_, we can easily obtain that [*x*]_*A*_^⪰^ = [*x*]_*B*_^⪰^ holds for any *x* and *D*_*j*_. Then |*D*_*j*_∩[*x*]_*A*_^⪰^ | = |*D*_*j*_∩[*x*]_*B*_^⪰^| holds for any *x* and *D*_*j*_. We can obtain that *μ*_*B*_^⪰^(*x*) = *μ*_*A*_^⪰^(*x*) holds for any *x*. That is, *B* is a distribution consistent set.

To acquire reductions in inconsistent ordered information system, the matrices can be the only forms of storage in computing. And we illustrate the progress to calculate the reductions as shown in Algorithm 1.

**Figure 1:**
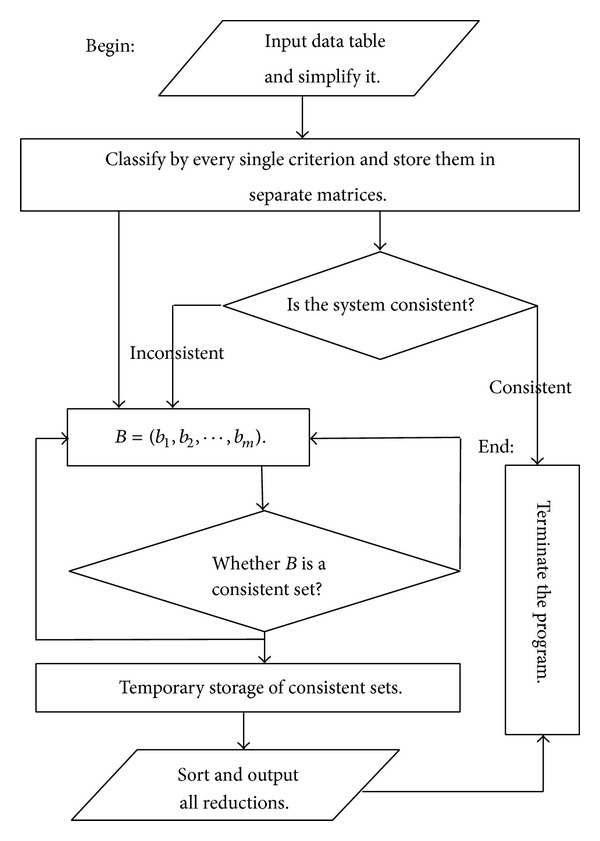
The flow chart of Algorithm 1.

The algorithm and the distribution reduction allow us to calculate reductions which keep the classification ability the same with the original system in a brief way. And we do not need to acquire every approximation of the decisions. It shortens the computing time and provides an effective tool for knowledge acquisition in criterion based rough set theory. The flow chart of the Algorithm 1 can be designed and it is placed in Figure 1. 

*Analysis to Time Complexity of Algorithm* 1. Let *I* ^⪰^ = (*U*, *A* ∪ *D*, *F*, *G*) be an ordered information system. *U* = {*x*_1_, *x*_2_,…, *x*_*n*_} is the simplified universe. The number of objects in original information system not being simplified is denoted by *n*_1_. There are *m* condition attributes in *A*; that is, |*A*| = *m*. The number of compressed decision classes is *r*. We take a variable *t*_*i*_ to stand for the time complexity in an implementation. In the next, we can analyze the time complexity of Algorithm 1 step by step.

The time complexity to simplify the original information system is *n*_1_^2^ for any two objects being compared and is denoted by *t*_1_ = *n*_1_^2^. Since |*U*| = *n*, |*A*| = *m*, and |*D*| = 1, the time complexities to be classified by condition attributes and decision *D* are, respectively, *t*_2_ = |*U*|^2^ × |*A*| and *t*_3_ = |*U*|^2^. For decision classes being merged by comparing classes of any two objects, the time complexity is *t*_4_ = |*U*|^2^. Now the consistency of the information system needs to be checked by comparing the condition class and decision class of any object. If the information system is consistent, the time complexity to check consistency is |*U*|. If the information system is inconsistent, the time complexity to check consistency is less than |*U*|. Thus, the time complexity to check consistency is no more than |*U*|; that is, it is presented as *t*_5_ ≤ |*U*|. Then, the possible and compatible distribution functions can be calculated and the time complexity is *t*_6_ = 2*r* × |*U*|. The time complexity to calculate each of these two functions is *r* × |*U*| and is denoted by *t*_6_^*σ*^ = *t*_6_^*δ*^ = *r* × |*U*|. The analysis to Step 1 is finished.

For Step 2, the time complexity to calculate possible and compatible distribution decision matrices, respectively, is denoted by *t*_7_^*σ*^ = *t*_7_^*δ*^ = |*U*|^2^. Thus, the time complexity to calculate distribution decision matrices is *t*_7_ = 2|*U* ^2^|. The time complexity of Step 2 is completed.

The first two steps are preparations to calculate reductions. The next Step 3 to Step 5 are the steps which run the operations. There are C_*m*_^1^ = *m* subsets {*a*_*l*_} and the dominance matrices are with dimensions *n* × *n*. In addition, the representation C_*m*_^*i*^ is the combinatorial number which means the number of selections to chose *i* elements from *m* ones. We consider that the judgement of a vector if it is zero runs one operation and the comparison of two vectors runs according to the dimension of the vectors. Therefore, the time complexities to compare *M*_*d*_^*σ*^ and *M*_*d*_^*δ*^ with *M*_{*a*_*l*_}_, respectively, are |*U*|^2^. And the time complexity to compare every line vector of *M*_{*a*_*l*_}_ with zero is |*U*|. The possible and compatible distribution matrices are obtained by reassignment values *n* times. And the time complexities to process possible and compatible distribution matrices, respectively, are both *n*. Then, we have that the total time complexity of Step 3 is *t*_8_ = C_*m*_^1^ × (3|*U*|^2^ + 3|*U*|). The judgement in Step 4 just need to run according to the number of {*a*_*l*_} and the time complexity is *t*_9_ = 2C_*m*_^1^.

Since we just need to compute the intersection of nonzero 1st order possible (or compatible) distribution matrices, the maximum time complexities can be analyzed in the next steps but not the true ones in computing. Therefore, the maximum time complexity relies on the number of attribute subsets 2^|*C*|^. The worst case is that no minimum reduction exists in the information system and all 2^|*C*|^ subsets are calculated in the algorithm. Thus, the maximum time complexity of Step 5 is *t*_10_ = 2C_*m*_^2^ × |*U*|^2^.

From the above analysis, we can know that the maximum time complexity of the main part in the algorithm (Step 3 to Step 5) is *t*_main_ = *t*_8_ + *t*_9_ + *t*_10_ = |*A*| × (|*U*|^2^ + |*U*|).

Hence, the maximum time complexity of the main algorithm is approximately *O*((|*U*|^2^ + 2|*U*|) × |*A*|).

## 5. Experimental Computing and Case Study

We design programs and employ two cases to demonstrate the effective of the method in the last section. This experimental computing program is running on a personal computer with the following hardware and software configuration. The configuration of the computer is a bit low but the program runs well and fast. It also shows the advantage of Algorithm 1 and the corresponding computing program (see Table 2).

An inconsistent ordered information system on animals sleep is presented in Table 3.

The information system is denoted by *I* ^⪰^ = (*U*, *A* ∪ {*d*}, *V*, *f*), where *A* is the condition attribute set and *d* is the single dominance decision. There are 42 objects which represent the species of animals and 10 attributes with numerical values in the ordered information system. The animals' names are showed in Table 3 and the interpretations of the attributes will be listed as follows. The interpretations and the units of attributes are represented as shown in Table 4.

By the experimental computing program, the distribution reductions of the system can be calculated and they are represented in the following. The operating time to compute this case is 0.158581 seconds.

The distribution reductions are

(11) $$\begin{array}{c} \left\{a_{1},a_{3},a_{4},a_{6},a_{7},a_{8},a_{9}\right\},\\ \left\{a_{2},a_{3},a_{4},a_{6},a_{7},a_{8},a_{9}\right\},\\ \left\{a_{1},a_{2},a_{3},a_{4},a_{6},a_{7},a_{8},a_{9}\right\},\\ \left\{a_{1},a_{3},a_{4},a_{5},a_{6},a_{7},a_{8},a_{9}\right\},\\ \left\{a_{2},a_{3},a_{4},a_{5},a_{6},a_{7},a_{8},a_{9}\right\}. \end{array}$$

And it can be verified by taking the computer as an assistant that the above sets are reductions of the data table. Detailed progress of the verifying are not arranged here. From the results, we can easily see that the reductions studied in this paper are different from the ones approached in since these reductions are {*a*_3_, *a*_4_, *a*_6_, *a*_7_}, {*a*_4_, *a*_5_, *a*_6_, *a*_7_}, {*a*_6_, *a*_8_, *a*_9_}, and {*a*_1_, *a*_2_, *a*_8_, *a*_9_}. They are different kinds of reductions in ordered information systems and can adapt to different needs in practices. From the definition of different reductions, we can also easily obtain that possible and compatible reductions are usually subsets of distribution reduction. This is not strict and should be studied and verified separately and theatrically. And the work may be taken into account as one part of the future studies in our work.

Finally, we take other inconsistent ordered information system to acquire the distribution reduction, respectively. And the descriptions on the data tables are listed in Table 5.

From the results in Table 5, we can obtain that the algorithm and the program we studied in this paper could be effective and useful to acquire distribution reductions in practice. The numbers of objects and attributes can increase the computing time. But the matrices storage has the ability to shorten the memory and computing time. And it can be helpful in research theoretically and it is applicable.

## 6. Conclusions

As is known, many information systems are data tables considering criteria for various factors in practise. Therefore, it is meaningful to study the attribute reductions in inconsistent information system on the basis of dominance relations. In this paper, distribution reduction is restated in inconsistent ordered information systems. Some properties and theorems are studied and discussed. A fact is certified that the distribution reduction is equivalent to the maximum distribution reduction in ordered information systems. Theorems on distribution reduction are implemented to create preparations for reduction acquisition and the dominance matrix is also restated to acquire distribution reductions in criterion based information systems. The matrix algorithm for distribution reduction acquisition is stepped and programmed. The algorithm can provide an approach and the program can be effective for theoretical research on knowledge reductions in criterion based inconsistent information systems. Dominance matrices are the only relied parameters which need to be considered without others such as approximations and subinformation systems being brought in. Furthermore, cases are employed to illustrate the validity of the matrix method and the program, which shows that the effectiveness of the algorithm in complicated information systems.

## Figures and Tables

**Algorithm 1:**
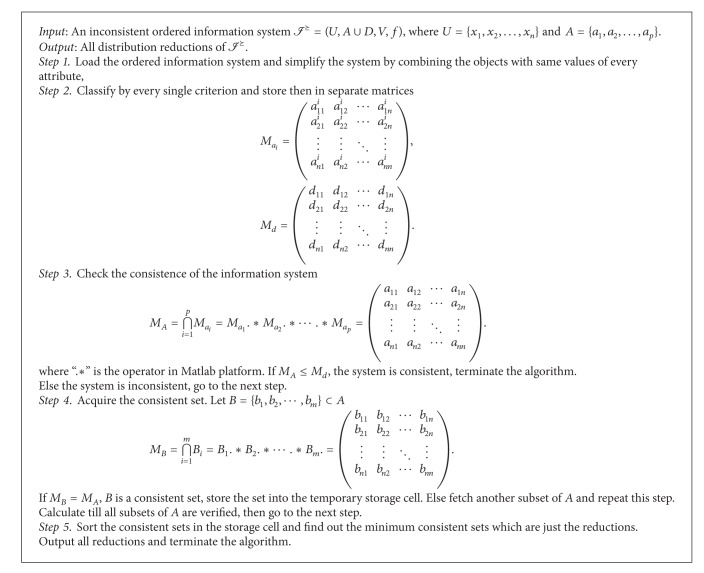


**Table 1:** An ordered information system.

*U*	*a*_1_	*a*_2_	*a*_3_	*d*
*x*_1_	1	2	1	3
*x*_2_	3	2	2	2
*x*_3_	1	1	2	1
*x*_4_	2	1	3	2
*x*_5_	3	3	2	3
*x*_6_	3	2	3	1

**Table 2:** 

Names	Models	Parameters
CPU	Intel Core i3-380U	1.33 GHz
Memory	DDR3 SDRAM	2 × 2 GB 1333 MHz
Hard disk	TOSHIBA	320 GB
System	Windows 7	32 bit
Platform	Matlab	Leasehold

**Table 3:** *I* ^⪰^: An inconsistent ordered information system on animals sleep.

	(*U*, *C* ∪ {*d*})	*a*_1_	*a*_2_	*a*_3_	*a*_4_	*a*_5_	*a*_6_	*a*_7_	*a*_8_	*a*_9_	*d*
*x*_1_:	African giant pouched rat	1	6.6	6.3	2	8.3	4.5	42	3	1	3
*x*_2_:	Asian elephant	2547	4603	2.1	1.8	3.9	69	624	3	5	4
*x*_3_:	Baboon	10.55	179.5	9.1	0.7	9.8	27	180	4	4	4
*x*_4_:	Big brown bat	0.023	0.3	15.8	3.9	19.7	19	35	1	1	1
*x*_5_:	Brazilian tapir	160	169	5.2	1	6.2	30.4	392	4	5	4
*x*_6_:	Cat	3.3	25.6	10.9	3.6	14.5	28	63	1	2	1
*x*_7_:	Chimpanzee	52.16	440	8.3	1.4	9.7	50	230	1	1	1
*x*_8_:	Chinchilla	0.425	6.4	11	1.5	12.5	7	112	5	4	4
*x*_9_:	Cow	465	423	3.2	0.7	3.9	30	281	5	5	5
*x*_10_:	Eastern American mole	0.075	1.2	6.3	2.1	8.4	3.5	42	1	1	1
*x*_11_:	Echidna	3	25	8.6	0	8.6	50	28	2	2	2
*x*_12_:	European hedgehog	0.785	3.5	6.6	4.1	10.7	6	42	2	2	2
*x*_13_:	Galago	0.2	5	9.5	1.2	10.7	10.4	120	2	2	2
*x*_14_:	Goat	27.66	115	3.3	0.5	3.8	20	148	5	5	5
*x*_15_:	Golden hamster	0.12	1	11	3.4	14.4	3.9	16	3	1	2
*x*_16_:	Gray seal	85	325	4.7	1.5	6.2	41	310	1	3	1
*x*_17_:	Ground squirrel	0.101	4	10.4	3.4	13.8	9	28	5	1	3
*x*_18_:	Guinea pig	1.04	5.5	7.4	0.8	8.2	7.6	68	5	3	4
*x*_19_:	Horse	521	655	2.1	0.8	2.9	46	336	5	5	5
*x*_20_:	Lesser short-tailed shrew	0.005	0.14	7.7	1.4	9.1	2.6	21.5	5	2	4
*x*_21_:	Little brown bat	0.01	0.25	17.9	2	19.9	24	50	1	1	1
*x*_22_:	Man	62	1320	6.1	1.9	8	100	267	1	1	1
*x*_23_:	Mouse	0.023	0.4	11.9	1.3	13.2	3.2	19	4	1	3
*x*_24_:	Musk shrew	0.048	0.33	10.8	2	12.8	2	30	4	1	3
*x*_25_:	N. American opossum	1.7	6.3	13.8	5.6	19.4	5	12	2	1	1
*x*_26_:	Nine-banded armadillo	3.5	10.8	14.3	3.1	17.4	6.5	120	2	1	1
*x*_27_:	Owl monkey	0.48	15.5	15.2	1.8	17	12	140	2	2	2
*x*_28_:	Patas monkey	10	115	10	0.9	10.9	20.2	170	4	4	4
*x*_29_:	Phanlanger	1.62	11.4	11.9	1.8	13.7	13	17	2	1	2
*x*_30_:	Pig	192	180	6.5	1.9	8.4	27	115	4	4	4
*x*_31_:	Rabbit	2.5	12.1	7.5	0.9	8.4	18	31	5	5	5
*x*_32_:	Rat	0.28	1.9	10.6	2.6	13.2	4.7	21	3	1	3
*x*_33_:	Red fox	4.235	50.4	7.4	2.4	9.8	9.8	52	1	1	1
*x*_34_:	Rhesus monkey	6.8	179	8.4	1.2	9.6	29	164	2	3	2
*x*_35_:	Rock hyrax (Hetero.b)	0.75	12.3	5.7	0.9	6.6	7	225	2	2	2
*x*_36_:	Rock hyrax (Procavia hab)	3.6	21	4.9	0.5	5.4	6	225	3	2	3
*x*_37_:	Sheep	55.5	175	3.2	0.6	3.8	20	151	5	5	5
*x*_38_:	Tenrec	0.9	2.6	11	2.3	13.3	4.5	60	2	1	2
*x*_39_:	Tree hyrax	2	12.3	4.9	0.5	5.4	7.5	200	3	1	3
*x*_40_:	Tree shrew	0.104	2.5	13.2	2.6	15.8	2.3	46	3	2	2
*x*_41_:	Vervet	4.19	58	9.7	0.6	10.3	24	210	4	3	4
*x*_42_:	Water opossum	3.5	3.9	12.8	6.6	19.4	3	14	2	1	1

**Table 4:** 

*a*_1_—Bodyweight in kg;	
*a*_2_—Brain weight in g;	
*a*_3_—Show wave (“nondreaming") sleep (hrs/day);	
*a*_4_—Paradoxical (“dreaming") sleep (hrs/day);	
*a*_5_—Total sleep (hrs/day);	
*a*_6_—Maximum life span (years);	
*a*_7_—Gestation time (days);	
*a*_8_—Predation index (1–5);	
*a*_9_—Sleep exposure index (1–5);	
*d*—Overall danger index (1–5).	

**Table 5:** Descriptions on the calculations.

Data name	Values	Objects	Conditions	Decisions	Reductions	Time	Operations
Body fat	Real	252	14	1	11	36.43723 s	10
Glass	Real	213	9	1	7	2.04624 s	10
Animal sleep	Real	42	9	1	5	0.13153 s	10
